# Leptin Administration Favors Muscle Mass Accretion by Decreasing FoxO3a and Increasing PGC-1α in *ob/ob* Mice

**DOI:** 10.1371/journal.pone.0006808

**Published:** 2009-09-04

**Authors:** Neira Sáinz, Amaia Rodríguez, Victoria Catalán, Sara Becerril, Beatriz Ramírez, Javier Gómez-Ambrosi, Gema Frühbeck

**Affiliations:** 1 Metabolic Research Laboratory, University of Navarra, Pamplona, Spain; 2 Department of Endocrinology, Clínica Universidad de Navarra, Pamplona, Spain; 3 CIBER Fisiopatología de la Obesidad y Nutrición (CIBEROBN), Instituto de Salud Carlos III, Madrid, Spain; University of Las Palmas de Gran Canaria, Spain

## Abstract

Absence of leptin has been associated with reduced skeletal muscle mass in leptin-deficient *ob/ob* mice. The aim of our study was to examine the effect of leptin on the catabolic and anabolic pathways regulating muscle mass. Gastrocnemius, extensor digitorum longus and soleus muscle mass as well as fiber size were significantly lower in *ob/ob* mice compared to wild type littermates, being significantly increased by leptin administration (*P*<0.001). This effect was associated with an inactivation of the muscle atrophy-related transcription factor forkhead box class O3 (FoxO3a) (*P*<0.05), and with a decrease in the protein expression levels of the E3 ubiquitin-ligases muscle atrophy F-box (MAFbx) (*P*<0.05) and muscle RING finger 1 (MuRF1) (*P*<0.05). Moreover, leptin increased (*P*<0.01) protein expression levels of peroxisome proliferator-activated receptor γ coactivator-1α (PGC-1α), a regulator of muscle fiber type, and decreased (*P*<0.05) myostatin protein, a negative regulator of muscle growth. Leptin administration also activated (*P*<0.01) the regulators of cell cycle progression proliferating cell nuclear antigen (PCNA) and cyclin D1, and increased (*P*<0.01) myofibrillar protein troponin T. The present study provides evidence that leptin treatment may increase muscle mass of *ob/ob* mice by inhibiting myofibrillar protein degradation as well as enhancing muscle cell proliferation.

## Introduction

Leptin, the product of the *ob* gene, is a hormone that acts as an afferent signal in a negative-feedback loop regulating the size of adipose tissue mass [1]. In addition to its function as a satiety factor, leptin regulates several physiological processes, such as glucose and lipid metabolism, immunity, reproduction and blood pressure homeostasis [2], [3]. Skeletal muscle also constitutes an important target for leptin [3], [4]. Several studies have reported that leptin-deficient *ob/ob* mice display a reduced skeletal muscle mass [5] compared with their lean littermates. However, the mechanisms whereby leptin regulates muscle growth are poorly understood.

Skeletal muscle mass and composition are critical for exercise, energy expenditure and glucose metabolism. Although the underlying mechanisms involved in the development of muscle atrophy are poorly understood, an imbalance between protein breakdown and synthesis, in favour of the former, plays an important role in this process [6]. The ubiquitin-proteasome system (UPS) is critical for the specific degradation of cellular proteins, being the main proteolytic system for protein breakdown in muscular atrophy. UPS induces the expression of the ubiquitin ligases E3 muscle atrophy F-box (MAFbx, also known as atrogin-1) and muscle RING finger-1 (MuRF1). These ubiquitin ligases target proteins with a ubiquitin chain to be subsequently degraded within the proteasome complex to peptides [7]. Both MAFbx and MuRF1 have been shown to be up-regulated in different models of atrophy, conferring them the status of muscle atrophy markers [8], [9]. Another key mediator in protein breakdown during atrophy is the forkhead box class O (FoxO) family of transcription factors [10]. In this sense, FoxO1 and FoxO3a, which are highly expressed in skeletal muscle, induce muscle mass loss by increasing the expression of MAFbx and MuRF1 [11]–[13], by inhibiting muscle growth and differentiation [14] and by impairing the progression of the cell cycle [15]. On the other hand, the enhancement of overall protein breakdown is blocked by the Akt signaling pathway. In its phosphorylated active form, Akt triggers the phosphorylation/inactivation of FoxO transcription factors, sequestering them to the cytosol, where they are unable to activate transcription of MAFbx and MuRF1 [16]. Nonetheless, under certain conditions, the regulation of Akt and FoxO seems to be independent of each other [17], [18]. Recently, it has been reported that peroxisome proliferator-activated receptor coactivator 1α (PGC-1α) also constitutes an important mediator of muscle mass down-regulating the expression levels and activity of FoxO3a and, hence, inhibiting muscle atrophy [19].

Obesity and insulin resistance exhibit a derangement in muscle mass regulation [6]. In this context, leptin has been shown to revert the obese and diabetic phenotype of *ob/ob* mice [20]. Thus, the aim of our study was to examine the catabolic and anabolic pathways involved in the regulation of muscle mass of *ob/ob* mice, and whether leptin administration normalizes the reduced skeletal muscle mass of leptin-deficient animals through FoxO-dependent mechanisms. Data of our study provide evidence that leptin treatment may increase muscle mass of *ob/ob* mice by inhibiting muscular atrophy markers as well as enhancing positive regulators of muscle cell proliferation.

## Results

### Leptin Administration Increases Muscle Mass

As expected, undetectable serum leptin concentrations were found in *ob/ob* mice. Leptin administration increased leptin levels in wild type and *ob/ob* mice. Importantly, the determination of leptin was performed 20 hours after the last exogenous administration of the hormone and measured values were whitin the nanomolar range observed under physiological circumstances. In addition, *ob/ob* mice used in our study were obese, hyperinsulinemic, hyperglycemic and hyperlipidemic. Leptin treatment corrected the obese phenotype and improved glucose and lipid metabolism of *ob/ob* mice, independently of the inhibitory effect of leptin on food intake, as compared to the pair-fed *ob/ob* group. Moreover, leptin also decreased body weight and fat mass of wild type mice independently of appetite reduction (Figure S1). The biochemical characteristics of wild type and *ob/ob* mice are reported in Table S1.

**Figure 1:**
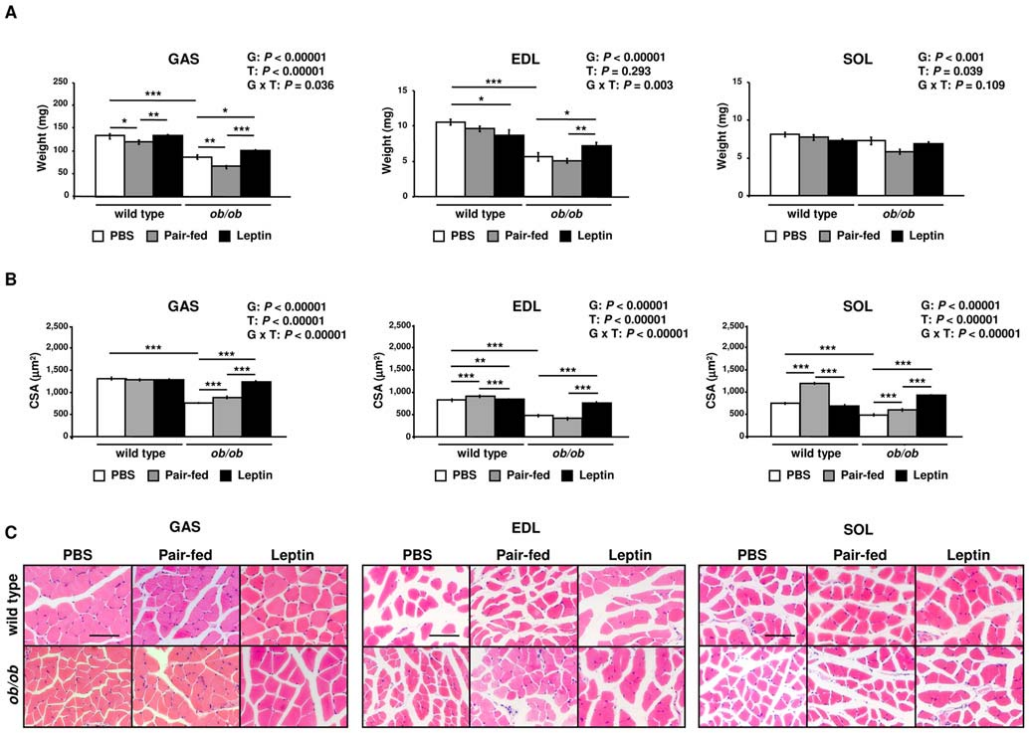
Leptin Increases Muscle Mass and Muscle Fiber Size of *ob/ob* Mice. (A) Gastrocnemius (GAS), extensor digitorum longus (EDL) and soleus (SOL) muscle weights of PBS (open), pair-fed (gray) and leptin-treated (closed) wild type and *ob/ob* mice (n = 9–10 per group). *P<0.05, **P<0.01 and ***P<0.001. (B) Cross-sectional area (CSA) of GAS, EDL and SOL muscle fibers of PBS (open), pair-fed (gray) and leptin-treated (closed) wild type and *ob/ob* mice (approximately 100 fibers/muscle from 3 mice/group). **P<0.001 and ***P<0.0001. (C) Representative histological sections of hematoxylin-eosin-stained GAS, EDL and SOL muscles of wild type and *ob/ob* mice. Scale bars = 200 µm. Results are presented as mean±SEM. G: genotype, T: treatment.

The weights of gastrocnemius (GAS) (*P*<0.0001), extensor digitorum longus (EDL) (*P*<0.0001) and soleus (SOL) (*P*<0.001) muscles were significantly lower in *ob/ob* mice as compared with *wild type*, showing a strong effect of leptin deficiency on muscle mass (Figure 1). In addition, pair-feeding decreased the weight of GAS in wild type (*P*<0.05) and *ob/ob* (*P*<0.01) mice. Leptin treatment significantly increased GAS and EDL mass compared to control (*P*<0.05) and pair-fed (*P*<0.01) obese animals and prevented the SOL mass loss induced by pair-feeding in *ob/ob* mice (*P*<0.05). Leptin administration also increased the weight of GAS muscle in wild type mice as compared to pair-fed animals (*P*<0.01). Curiously, the hormone reduced the weight of EDL in wild type mice (*P*<0.05). The cross-sectional area (CSA) of GAS, EDL and SOL muscle fibers was also decreased in *ob/ob* compared to wild type animals (*P*<0.0001), a condition that was completely reverted by leptin treatment (*P*<0.0001). Surprisingly, pair-feeding increased CSA of EDL and SOL muscle fibers in wild type mice (*P*<0.0001), which was not observed in leptin-treated wild type mice. Therefore, our data show that leptin administration increases muscle mass and muscle fiber size of *ob/ob* mice.

### Gene Expression Profiles Reveal Significant Changes in Skeletal Muscle after Leptin Treatment

To investigate the effect of leptin on the catabolic and anabolic pathways involved in the regulation of the GAS muscle of wild type and *ob/ob* mice, their differential gene expression profiles were analyzed by microarray analysis. The hierarchical clustering of the gene expression profile in GAS muscle of wild type, *ob/ob* and leptin-treated *ob/ob* mice is shown in Figure S2. Microarray data showed 1,127 genes with differential expression (1.5-fold change) for leptin deficiency (wild type *vs ob/ob*; 51.5% up-regulated and 48.5% down-regulated genes), 1,546 genes for leptin administration (*ob/ob vs* leptin-treated *ob/ob* mice; 33.1% up-regulated and 66.9% down-regulated genes), and 1,960 for appetite inhibiting-independent effects of leptin (pair-fed *ob/ob vs* leptin-treated *ob/ob* group; 50.2% up-regulated and 49.8 down-regulated genes). The set of genes with altered expression levels induced by leptin deficiency and administration represents a broad spectrum of biological processes involved in muscle growth and atrophy, such as cell cycle progression, ubiquitin-proteolysis and apoptosis (Tables S2 and S3 in Supporting Information). Our study shows an up-regulation of positive regulators of muscle growth and cell cycle progression in GAS muscle of leptin-treated *ob/ob* mice (*Igf1, Igfbp5, Notch3, Ccnd1, Clk4, Cited4, Cdc14a*), and myoblast differentiation (*Eya1*, *Mkl1*, *Srf*), whereas negative regulators of cell cycle progression, such as *Cdkn1a/p21*, C*dkn1b/p27^Kip1^* or *Rbl2*, were down-regulated by leptin administration in *ob/ob* mice. Moreover, leptin treatment down-regulated the expression levels of positive regulators of ubiquitin proteolysis (*Fbxo32/MAFbx, Need4, Ube2h, Ub1x*), apoptosis (*Acin1, Amid, Dffa, Bclaf1*) and autophagy (*Lysmd3, Becn1, Atg121*) and up-regulated inhibitory factors of apoptosis (*Apip, Bcl2*) (Tables 1 and S2). Leptin administration also increased the gene expression levels of positive regulators of protein synthesis (*Eif4e, Eefe1*) and decreased the mRNA expression of inhibitors of protein synthesis (*Gsk3b, Pten*). Increased gene expression levels codifying for the contractile and sarcomeric proteins myosin (*Myh1/Mhc2x*, *Myh2/Mhc2a*, *Myh7/Mhc1*, *Myl3, Myl4*), troponin (*Tnni1*, *Tnnt1*, *Tnnc1*), tropomyosin (*Tpm3*), nebulin (*Neb*) and titin (*Ttn*) were normalized by leptin treatment in *ob/ob* mice (Tables 1 and S2). Furthermore, leptin prevented the up-regulation of positive regulators of ubiquitin proteolysis (*Trim63/MuRF1, Fbxo32/MAFbx, Foxo1*) and autophagy (*Gabarap11*) induced by pair-feeding in *ob/ob* mice (Table S3). Microarray data evidenced that leptin is an important regulator of the expression of genes involved in muscle growth and muscle atrophy. To confirm the microarray data, the mRNA expression of a number of representative transcripts involved in muscle growth and atrophy was analyzed by Real-Time PCR (Figure S3).

**Table 1:** Selected Genes Regulated by Leptin in Gastrocnemius Muscle.

Gene Ontology Biological Process	GeneBank Number	Gene Symbol	Gene name	Fold change
**Up-regulated genes**				
**GO: 0007049. Cell cycle**				
**Positive regulators of cell cycle progression**				
NM_007631		***Ccnd1***	Cyclin D1	1.217
XM_149387		***Cdc14a***	CDC14 cell division cycle 14 homolog A isoform 1	2.717
NM_019563		***Cited4***	Cbp/p300-interacting transactivator, with Glu/Asp-rich carboxy-terminal domain, 4	2.251
NM_007714		***Clk4***	CDC like kinase 4	1.503
NM_010512		***Igf1***	Insulin-like growth factor 1 isoform 1	1.394
NM_010518		***Igfbp5***	Insulin-like growth factor binding protein 5	1.820
**GO: 0019538. Protein metabolic process**				
**Positive regulators of Protein synthesis**				
NM_025380		***Eef1e1***	Eukaryotic translation elongation factor 1 ε 1	1.359
NM_007917		***Eif4e***	Eukaryotic translation initiation factor 4E	1.454
**GO: 45445. Myoblast differentiation**				
**Positive regulators of cell differentiation**				
BC060260		***Eya1***	Eyes absent 1 homolog	1.637
AK044188		***Mkl1***	Myocardin-like 1	1.802
NM_020493		***Srf***	Serum response factor	1.311
**GO: 6915. Apoptosis**				
**Negative regulators of apoptosis**				
NM_019735		***Apip***	APAF1 interacting protein	1.282
NM_009741		***Bcl2***	B-cell leukemia/lymphoma 2 isoform 1	1.475
**Down-regulated genes**				
**GO: 0007049. Cell cycle**				
**Negative regulators of cell cycle**				
NM_007669		***Cdkn1a***	Cyclin-dependent kinase inhibitor 1A (P21)	0.612
NM_009875		***Cdkn1b***	Cyclin-dependent kinase inhibitor 1B (P27)	0.472
AK077477		***Igfbp3***	Insulin-like growth factor binding protein 3	0.542
NM_011250		***Rbl2***	Retinoblastoma-like 2	0.631
**GO: 0006914. Autophagy**				
**Positive regulators of autophagy**				
NM_026217		***Atg12l***	Autophagy-related 12-like	0.814
NM_030257		***Lysmd3***	LysM, putative peptidoglycan-binding, domain containing 3	0.655
NM_019584		***Becn1***	Beclin 1	0.802
**GO: 6915. Apoptosis**				
**Positive regulators of apoptosis**				
NM_023190		***Acin1***	Apoptotic chromatin condensation inducer 1 isoform 2	0.502
NM_153779		***Amid***	Apoptosis-inducing factor (AIF)-like mitchondrion-associated inducer of death isoform 1	0.658
NM_007523		***Bak1***	BCL2-antagonist/killer 1	0.550
NM_153787		***Bclaf1***	BCL2-associated transcription factor 1 isoform 2	0.543
NM_001025296		***Dffa***	DNA fragmentation factor, α subunit isoform a	0.484
**GO: 6511. Ubiquitin-dependent protein catabolism**				
**Positive regulators of ubiquitin-proteolysis**				
NM_026346		***Fbxo32***	F-box only protein 32	0.666
NM_010890		***Nedd4***	Neural precursor cell expressed, developmentally down-regulated gene 4	0.507
NM_009457		***Ube1x***	Ubiquitin-activating enzyme E1, Chr X	0.414
NM_009459		***Ube2h***	Ubiquitin-conjugating enzyme E2H	0.403
**GO: 0019538. Protein metabolic process**				
**Negative regulators of Protein synthesis**				
NM_010124		***Eif4ebp2***	Eukaryotic translation initiation factor 4E binding protein 2	0.871
NM_008960		***Pten***	Phosphatase and tensin homolog	0.665
NM_019827		***Gsk3b***	Glycogen synthase kinase 3β	0.464
**GO: 45214. Sarcomere organization**				
**Contractile and sarcomeric proteins**				
XM_354615		***Myh1***	Myosin, heavy polypeptide 1, skeletal muscle, adult	0.521
NM_144961		***Myh2***	Myosin, heavy polypeptide 2	0.521
XM_354614		***Myh3***	Myosin, heavy polypeptide 3, skeletal muscle, embryonic	0.214
NM_080728		***Myh7***	Myosin, heavy polypeptide 7, cardiac muscle β	0.665
NM_010859		***Myl3***	Myosin, light polypeptide 3	0.585
NM_010858		***Myl4***	Myosin, light polypeptide 4	0.688
XM_130232		***Neb***	Nebulin	0.386
NM_021467		***Tnni1***	Troponin I, skeletal, slow 1	0.438
NM_011618		***Tnnt1***	Troponin T1, skeletal, slow	0.687
NM_022314		***Tpm3***	Tropomyosin 3γ	0.648

Fold changes between *ob/ob vs* leptin-treated *ob/ob* mice of selected differentially expressed genes from Table S2.

### Leptin Administration Protects from Muscular Atrophy

No differences in the transcript levels of *Foxo1* and *Foxo3a* in GAS muscle were detected between wild type and *ob/ob* mice, but a tendency towards a down-regulation of both transcription factors was found after leptin treatment (Figure S3).

**Figure 2:**
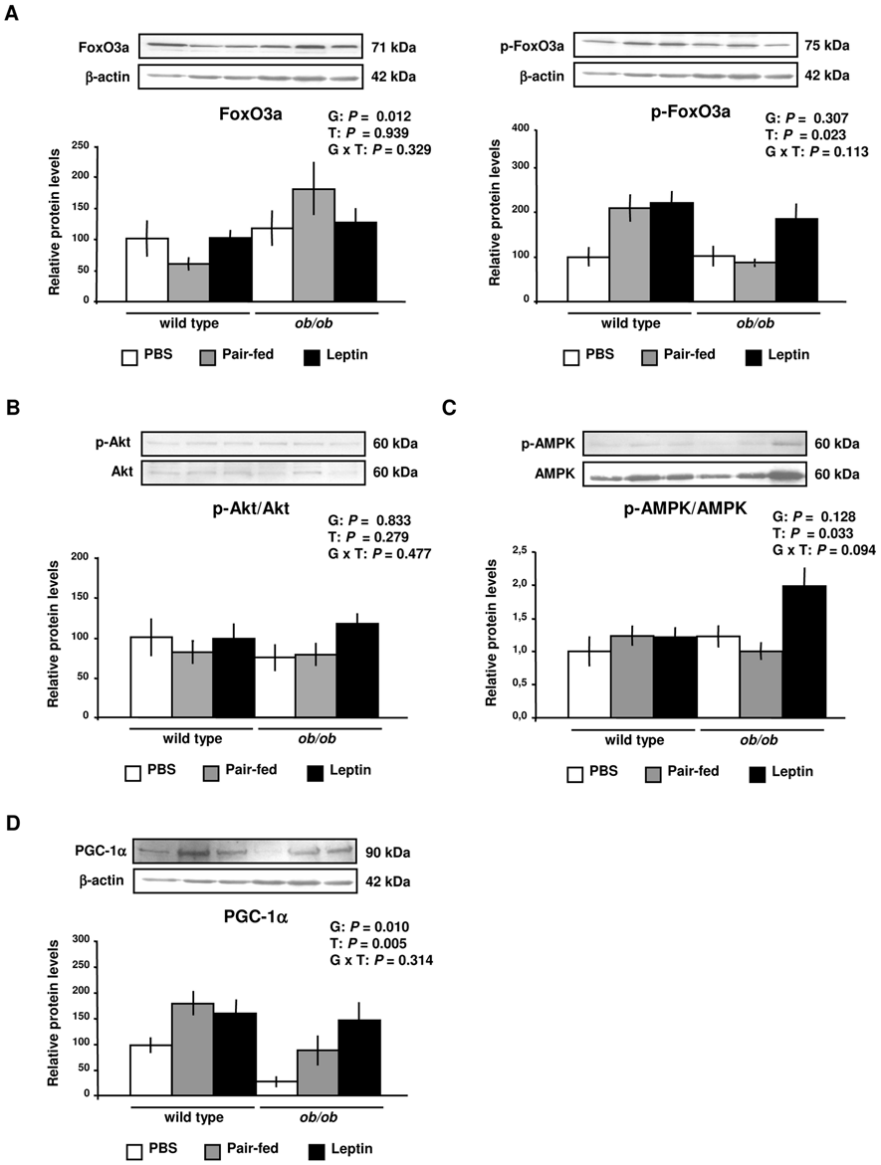
Leptin Decreases FoxO3a Activity and Increases PGC-1α Protein in Gastrocnemius Muscle. (A) Representative Western blot analyses of forkhead box class O (FoxO3a) and phospho-FoxO3a proteins of gastrocnemius muscle of PBS (open), pair-fed (gray) and leptin-treated (closed) wild type and *ob/ob* mice are shown. β-actin was used as a loading control (n = 8 per group). (B) Representative Western blot analyses of Akt activity evidenced by phosphorylated-Akt and Akt proteins ratio of gastrocnemius muscle of PBS (open), pair-fed (gray) and leptin-treated (closed) wild type and *ob/ob* mice (n = 8 per group). (C) Representative Western blot analyses of AMP-activated protein kinase (AMPK) activity evidenced by phosphorylated-AMPK and AMPK proteins ratio of gastrocnemius muscle of PBS (open), pair-fed (gray) and leptin-treated (closed) wild type and *ob/ob* mice are shown (n = 5 per group). (D) Representative Western blot analyses of AMP- (PGC-1α) of gastrocnemius muscle of PBS (open), pair-fed (gray) and leptin-treated (closed) wild type and *ob/ob* mice are shown. β-actin was used as a loading control (n = 8 per group). Data are expressed as mean±SEM. G: genotype, T: treatment.

Nonetheless, at the protein level, the active form of FoxO3a was increased (*P* = 0.012) in *ob/ob* mice, with the phosphorylated-FoxO3a (inactive form) being increased by leptin administration (*P* = 0.023) in wild type and *ob/ob* mice (Figure 2A). Thus, since the activity of FoxO3a is inhibited by phosphorylation, data show that leptin reduces the functional form of this transcription factor in GAS muscle of wild type and *ob/ob* mice. No effect of genotype (*P* = 0.833) or leptin treatment (*P* = 0.279) was observed on the activity of Akt in GAS muscle of experimental animals (Figure 2B). On the contrary, the activity of the AMP-activated protein kinase (AMPK) was increased by leptin treatment in *ob/ob* mice (*P* = 0.033) (Figure 2C). Gene expression studies showed a significant decrease (*P*<0.001) in *Pgc-1α* mRNA levels in GAS muscle of *ob/ob* mice (Figure S3). The effect of leptin deficiency on PGC-1α protein expression showed a similar pattern, being lower in *ob/ob* mice compared to wild type mice (*P* = 0.010). However, protein expression levels of PGC-1α were significantly increased (*P* = 0.005) by leptin administration in skeletal muscle of wild type and *ob/ob* mice (Figure 2D).

**Figure 3:**
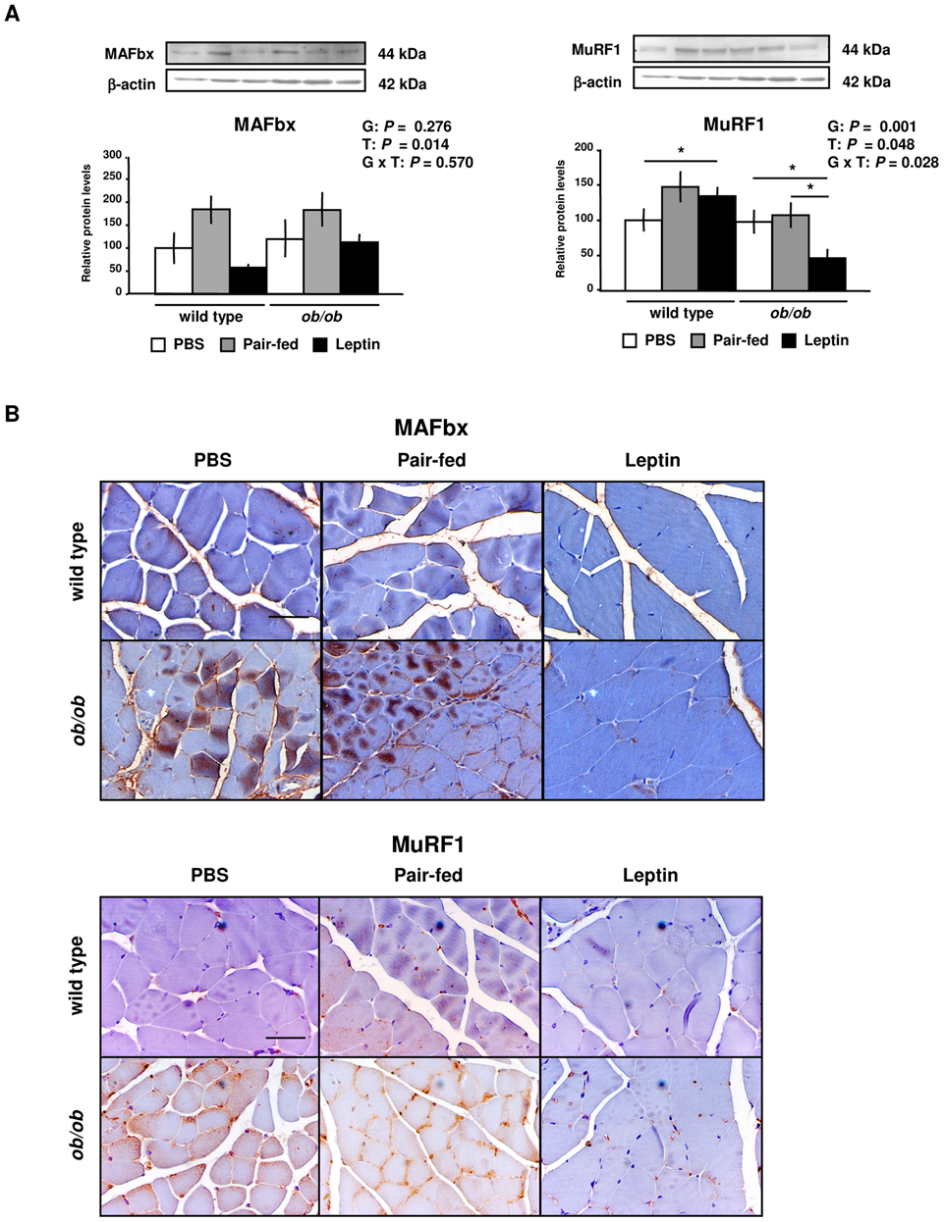
Leptin Decreases MAFbx and MuRF1 Protein Expression in Gastrocnemius Muscle of Wild Type and *ob/ob* Mice. (A) Representative Western blot analyses of muscle atrophy F box (MAFbx) and muscle RING finger 1 (MuRF1) proteins of gastrocnemius muscle of PBS (open), pair-fed (gray) and leptin-treated (closed) wild type and *ob/ob* mice are shown. β-actin was used as a loading control (n = 8 per group). *P<0.05. (B) Immunostaining for MAFbx and MuRF1 proteins was assessed by optic microscopy. Representative images are shown for gastrocnemius muscle. Scale bars = 50 µm. Data are presented as mean±SEM. G: genotype, T: treatment.

Leptin treatment decreased the mRNA expression levels of the atrophy marker MAFbx (*P* = 0.006) and tended to decrease MuRF1 in *ob/ob* mice, although no significant differences were found for the global effect of treatment (Figure S3 and Table S3). The protein content of MAFbx (*P* = 0.014) and MuRF1 (*P* = 0.021) was also reduced by leptin treatment in GAS muscle of wild type and *ob/ob* mice (Figure 3A). Since MAFbx and MuRF1 proteins have been associated with myofibrillar protein degradation, their tissue distribution was analyzed by immunohistochemical analysis. Fiber sections of GAS muscle showed higher sarcolemmal, cytosolic and nuclear immunoreactivity of MAFbx and MuRF1 in *ob/ob* mice as compared to wild type mice and, analogously to what happened with the protein content, were reduced in the leptin-treated *ob/ob* group (Figure 3B). Taken together, these results suggest that leptin administration decreases protein expression levels of the atrophy markers MAFbx and MuRFa in GAS muscle of wild type and *ob/ob* mice.

### Leptin Treatment Enhances Skeletal Muscle Growth

**Figure 4:**
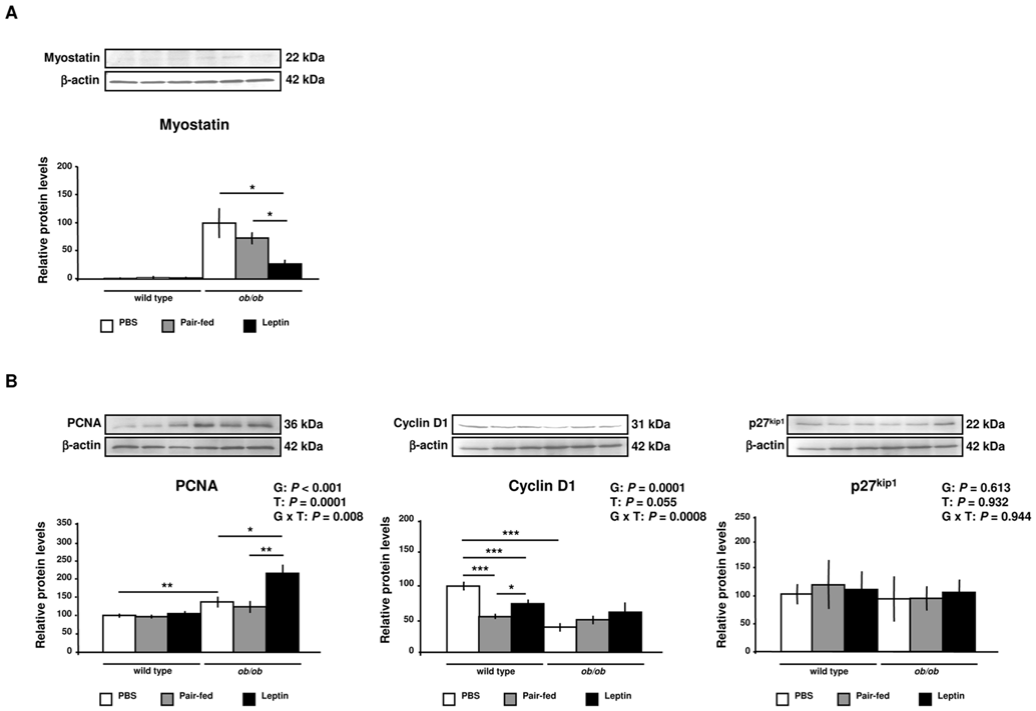
Leptin Regulates Muscle Growth and Cell Cycle Factors. (A) Representative Western blot analyses of myostatin protein of gastrocnemius muscle of PBS (open), pair-fed (gray) and leptin-treated (closed) wild type and *ob/ob* mice are shown. β-actin was used as a loading control (n = 2–6 per group). The myostatin band was detected only in 2 samples of wild type groups.*P<0.05. (B) Representative Western blot analyses of proliferating cell nuclear antigen (PCNA), cyclin D1 and cyclin-dependent kinase inhibitor 1B (Cdkn1b/p27^Kip1^) proteins of gastrocnemius muscle of PBS (open), pair-fed (gray) and leptin-treated (closed) wild type and *ob/ob* mice are shown. β-actin was used as a loading control (n = 8 per group). *P<0.05, **P<0.01 and ***P<0.001. Data are expressed as mean±SEM. G: genotype, T: treatment.

DNA microarray screening showed that leptin administration in *ob/ob* mice up-regulates genes involved in muscle growth and cell cycle in the GAS muscle (Table S2). We examined the effect of leptin on the protein expression of myostatin, a member of the transforming growth factor TGFβ family, which acts as a negative regulator of muscle growth [21]. The mature form of myostatin (22 kDa) was almost undetectable in wild type mice. However, data showed a high protein expression of myostatin in *ob/ob* mice, which was reduced after leptin treatment as compared to the *ob/ob* (*P* = 0.026) and pair-fed *ob/ob* mice (*P* = 0.015) (Figure 4A), suggesting that leptin enhances muscle growth in *ob/ob* mice.

**Figure 5:**
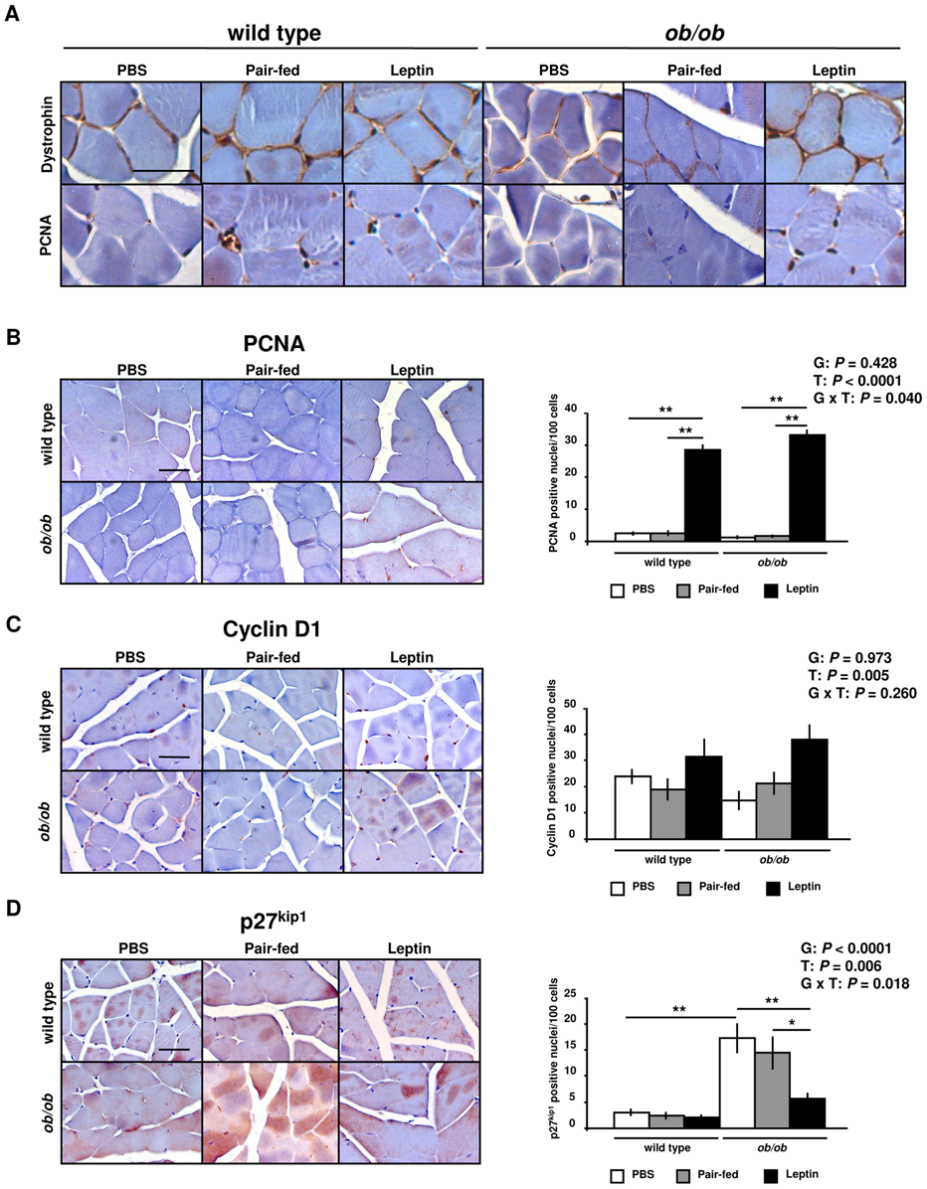
Leptin Enhances Muscle Cell Proliferation. (A) Immunohistochemical staining for dystrophin and PCNA of gastrocnemious muscle of wild type and *ob/ob* mice (n = 3 per group). Scale bar = 50 µm. (B) Immunohistochemical staining and relative stained nuclei number for PCNA evaluated among 500 cells in gastrocnemius muscle of PBS (open), pair-fed (gray) and leptin-treated (closed) wild type and *ob/ob* mice (n = 3 per group). Scale bar = 50 µm. **P<0.01. (C) Immunohistochemical staining and relative stained nuclei number for cyclin D1 evaluated among 500 cells in gastrocnemius muscle of PBS (open), pair-fed (gray) and leptin-treated (closed) wild type and *ob/ob* mice (n = 3 per group). Scale bar = 50 µm. (D) Immunohistochemical staining and relative stained nuclei number for p27^Kip1^ evaluated among 500 cells in gastrocnemius muscle of PBS (open), pair-fed (gray) and leptin-treated (closed) wild type and *ob/ob* mice (n = 3 per group). Scale bar = 50 µm. *P<0.05 and **P<0.01. Data are expressed as mean±SEM. G: genotype, T: treatment.

Myofibers are postmitotic muscle cells being unable to proliferate. Therefore, postnatal muscle growth depends on satellite cells, which are localized between the basal lamina and the plasmatic membrane of muscle fibers. Satellite cells are usually quiescent but are able to proliferate in response to mitogenic factors [22], [23]. The initiation of the cell cycle depends on the activity of the complexes of cyclin and cyclin-dependent kinases (CDK), while FoxO transcription factors inhibit the cell cycle progression of skeletal muscle by inducing CDK inhibitors [15]. The *ob/ob* mice showed an increase in the proliferating cell nuclear antigen (PCNA) (*P* = 0.010), a marker molecule for proliferating satellite cells [24], [25], and a decrease in cyclin D1 (*P* = 0.0001) proteins (Figure 4B). Interestingly, leptin treatment further increased PCNA as compared to the *ob/ob* (P = 0.021) and pair-fed *ob/ob* (*P* = 0.003) groups and tended to increase cyclin D1 protein in *ob/ob* mice (*P* = 0.055). No significant differences were found in the protein expression of the CDK inhibitor p27^Kip1^ (Figure 4B). Given that these regulators of the cell cycle are post-transcriptionally regulated by phosphorylation, being active within the nuclei [26]–[28], immunohistochemical analyses in sections of GAS muscle were performed to discriminate nuclear and cytosolic protein expression. In addition, we assessed immunostaining for the dystrophin protein, a marker molecule for plasmatic membrane (Figure 5A), to define positive PCNA nuclei and discriminate satellite cells from other cell types. In this sense, only nuclei between the basal lamina and the plasmatic membrane were counted. Leptin administration increased the nuclear immunostaining for PCNA (*P* = 0.002) (Figure 5B) and cyclin D1 (*P* = 0.005) (Figure 5C) in wild type and *ob/ob* mice. Moreover, nuclei of *ob/ob* mice showed an increased immunostaining of p27^Kip1^ (*P* = 0.002), which was reduced by leptin treatment as compared to *ob/ob* (*P* = 0.004) and pair-fed *ob/ob* (*P* = 0.015) mice (Figure 5D). These data provide evidence that leptin may enhance cell cycle progression in the GAS muscle of *ob/ob* mice.

**Figure 6:**
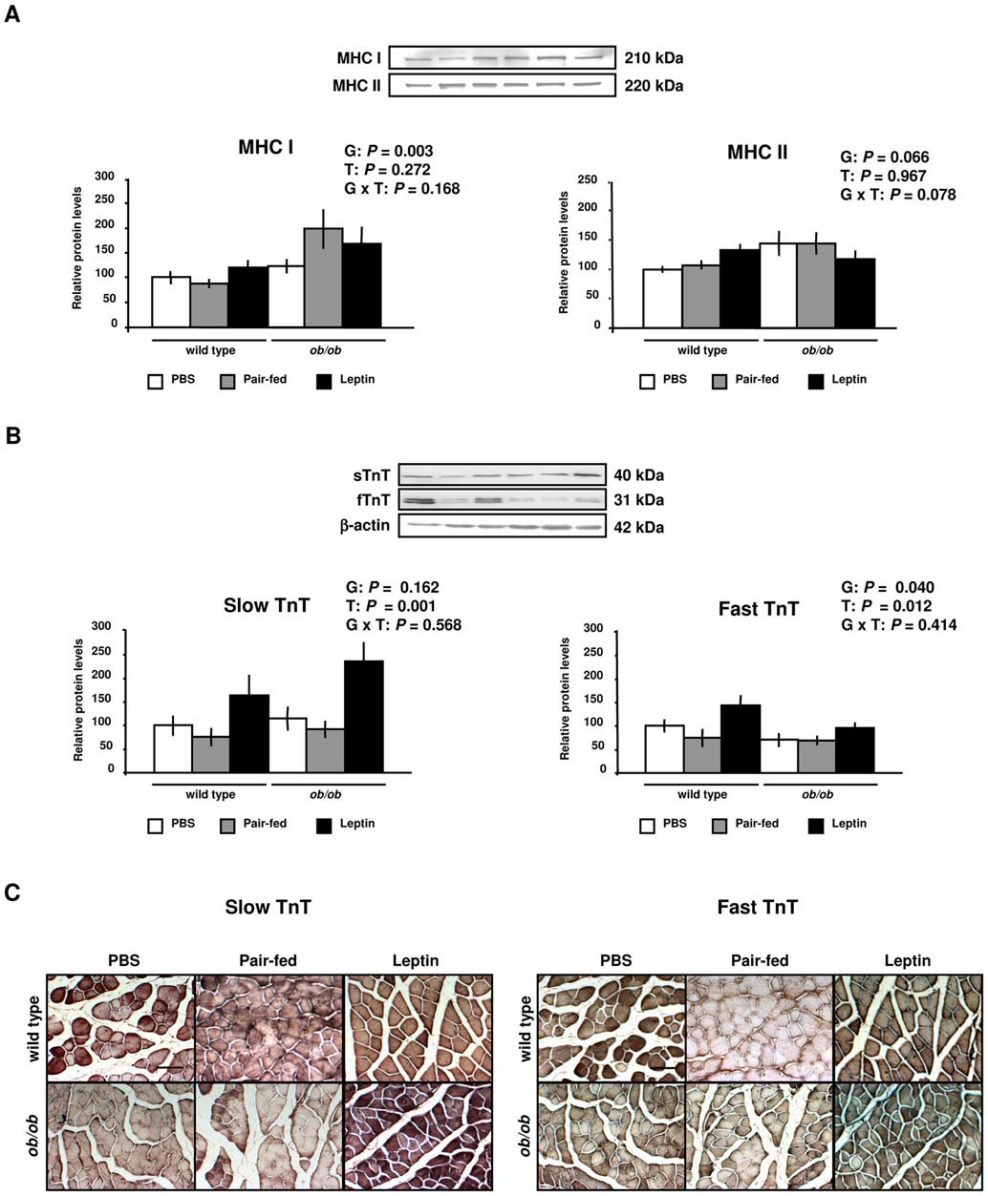
Leptin Increases the Expression of Contractile Proteins in Skeletal Muscle. (A) Representative Western blot analyses of myosin heavy chain type I (MHC I), myosin heavy chain type II (MHC II), slow and fast troponin T (TnT) proteins of gastrocnemius muscle of PBS (open), pair-fed (gray) and leptin-treated (closed) wild type and *ob/ob* mice are shown. β-actin was used as a loading control (n = 8 per group). (B) Immunohistochemical staining for slow and fast troponin T (TnT) of gastrocnemius muscle of wild type and *ob/ob* mice. Scale bars = 100 µm. Data are expressed as mean±SEM. G: genotype, T: treatment.

The growth of skeletal muscle requires an increase in the number of myonuclei together with an increase in the synthesis of myofibrillar proteins to maintain a constant ratio between number of nuclei per fiber and fiber size [29], [30]. Gene expression analyses showed up-regulation of genes codifying for contractile and sarcomeric proteins in the GAS muscle of *ob/ob* mice, which was normalized by leptin administration (Figure S3 and Table S2). Moreover, although a positive regulation of genes involved in the synthesis of proteins was induced by leptin administration (Table S2), no effect on the protein expression of both isoforms of MHC was found (Figure 6A). However, the administration of the hormone significantly increased the slow and fast isoforms of TnT in the GAS muscle of wild type and *ob/ob* mice (Figure 6B). These data were also confirmed by immunohistochemistry (Figure 6C). Therefore, leptin increases the protein expression of slow and fast isoforms of the contractile protein TnT.

## Discussion

Muscle loss is the result of a reduced protein synthesis and increased myofibrillar degradation in response to inactivity, food deprivation or catabolic diseases [6], [31]. Leptin-deficient *ob/ob* mice display a reduced skeletal muscle mass [5], [32], [33] and an increased muscle proteosome activity [34] compared to their lean counterparts. Moreover, leptin administration has been shown to inhibit protein breakdown in C2C12 myotubes [35]. However, other authors failed to observe any direct effect of leptin on the synthesis and degradation rates of proteins in skeletal muscle of rats [36], suggesting that leptin effects on muscle mass may be mediated by the release of a secondary growth factor. Our study provides evidence for a myogenic effect of leptin on wild type and *ob/ob* mice. The main findings reported herein are that: 1) leptin induces changes in the expression of transcription factors involved in muscle growth (reduced FoxO3a and increased PGC-1α); 2) leptin down-regulates the atrophy markers, MAFbx and MuRF1, as well as the negative regulator of muscle growth myostatin; and 3) leptin increases the positive cell cycle markers cyclin D1 and PCNA.

### Leptin Inhibits Muscular Atrophy

Animal and human studies have shown that caloric restriction enhances catabolic pathways in skeletal muscle through the activation of the energy sensor AMPK, which activates FoxO transcription factors leading to the up-regulation of MAFbx and MuRF1 [37]–[39]. Our results demonstrate that leptin increases the activity of AMPK in GAS of *ob/ob* mice as well as decreases the protein expression of FoxO3a and the mRNA and protein expression of MAFbx and MuRF1 in wild type and *ob/ob* mice. These data suggest that leptin treatment inhibits the catabolic pathway of proteins mediated by the AMPK. Overexpression of FoxO3a is sufficient to cause skeletal muscle atrophy by increasing the UPS activity [11], [14], but recent *in vivo* and *in vitro* studies have shown that FoxO3a also activates the autophagic/lysosomal pathway [12], [40]. In this sense, microarray data evidenced that *ob/ob* mice presented an upregulation of proteolytic (*Need4, Ube2h*), apoptotic (*Acin, Amid*), and autophagic (*Lysmd3, Becn1, Atg121*) genes, which were down-regulated by leptin treatment.

The activation of PI3K/Akt signaling by insulin and growth factors regulates muscular mass by reducing FoxO3a activity and, hence, by blocking the expression of MAFbx and MuRF1 [16]. Nonetheless, under certain conditions, regulation of Akt and FoxO seems to be independent of each other [17], [18]. Short-term leptin treatment activates Akt in smooth [41] and skeletal [42] muscles of rats, but long-term leptin treatment fails to activate this signaling pathway in myotubes [43] and in rat GAS muscle [44]. Our results showed that chronic leptin administration had no effect on Akt activity, which is in accordance with previous studies [43], [44].

PGC-1α protects skeletal muscle from atrophy by blocking FoxO3a action and atrophy-specific gene transcription [19]. The transcript levels of PGC-1α are dramatically decreased in models of mice with muscle atrophy induced by denervation and diabetes [45], while energy restriction enhances the expression of PGC-1α in skeletal muscle of rodents and humans [46]–[48]. Leptin-deficient *ob/ob* mice show a markedly reduced expression of *Pgc-1α* in brown adipose tissue, which is reverted by leptin treatment [49]. In the present study, we found that leptin deficiency is also associated with decreased PGC-1α expression in GAS muscle, and that leptin treatment significantly increased the PGC-1α protein in GAS muscle of wild type and *ob/ob* mice, which may be mediated by AMPK. In fact, AMPK reportedly enhances *Pgc-1α* up-regulation in muscle [50], [51] and AICAR, an AMPK-activating agent, leads to marked increases in PGC-1α protein content [52], [53]. Taken together, our findings suggest that leptin inhibits the pathways leading to muscle atrophy by increasing PGC-1α, which, in turn, decreases the expression of FoxO3a and its down-stream effectors, MAFbx and MuRF1 in GAS muscle of wild type and *ob/ob* mice.

### Leptin Enhances Muscle Growth

Muscle growth is related to a muscle fiber size increase, which involves a higher content of myofibrillar proteins as well as number of myonuclei to maintain a constant ratio between myonuclei per fiber and fiber size [30]. Mitogenic factors enhance cell cycle progression by increasing the activity of cyclin/CDK complexes, and inducing nuclear to cytoplasmic translocation of these inhibitors of cell cycle progression [23]. Moreover, FoxO proteins also regulate muscular mass by blocking the proliferation of muscle precursor cells via inhibiting DNA replication and cell cycle progression through the increase of the negative regulator of the cell cycle p27^Kip1^ [15], [54]. In this regard, our data show for the first time that leptin enhances cell cycle progression in GAS muscle by increasing the activity of the positive regulators of the cell cycle cyclin D1 and PCNA, and by simultaneously decreasing p27^Kip1^. These findings are in accordance with the mitogenic effects described for leptin in vascular endothelial cells [55], hepatocytes [56] cardiomyocytes [57], vascular smooth muscle cells [58], and myoblasts [59]. Until now, there is little information available on the effect of leptin on muscle growth *in vivo*. The work published by Ruth Harris [60], [61] and by Abram M. Madiehe et al. [60], [61] points to an increased protein synthesis and muscle mass in some strains of *db/db* mice suggesting that leptin is able to increase the growth of *db/db* mice via a mechanism that is dependent on the short isoform of the leptin receptor.

In order to explore other potential hormonal effects, circulating concentrations of testosterone were assessed taking into account that androgenic hormones affect muscle mass. However, no significant differences were observed following leptin administration. These data seem to indicate that the leptin effects on muscle mass observed in the present study are not related to the changes in testosterone levels. Our findings do not rule out the possibility that other hormones may be involved in the regulation of muscle mass of *ob/ob* mice. It is possible that changes observed in the leptin-treated mice are related to the overall hormonal changes elicited by the administration of leptin rather than due to the direct effects of leptin on muscle mass. In addition, physical activity is a key regulator for muscle mass and leptin reportedly increases the reduced spontaneous locomotor activity of *ob/ob* mice [62], [63]. Therefore, the possibility that muscle mass in leptin-treated *ob/ob* mice may be affected by physical activity can not be ruled out. Unfortunately, physical activity was not assessed in the present study.

It has been suggested that myostatin plays a direct role in the deterioration of the skeletal muscle in states of obesity and insulin resistance [64]–[66]. The biological action of myostatin is well described in myostatin-deficient mice, which exhibit muscular hyperplasia and hypertrophy [21], [67]. On the contrary, transgenic mice over-expressing myostatin exhibit a reduced muscular mass and fiber size [68], [69], which has lead to consider myostatin as a negative regulator of skeletal muscle growth. Interestingly, myostatin regulates muscle growth by inhibiting the proliferation [70], [71] and differentiation [72], [73] of myoblasts. Myostatin inhibits the progression from the G1 to the S phase in myoblasts [74], blocks the expression of cyclin D1 and induces its degradation by the UPS [75], [76]. In this respect, a high protein expression of myostatin was observed in GAS of *ob/ob* mice in the present study. Importantly, our data show that leptin decreases the high myostatin protein expression related to leptin deficiency in *ob/ob* mice, suggesting that leptin enhances muscle growth. These data are consistent with the inhibitory effect of leptin on the high mRNA myostatin levels in adipose tissue and skeletal muscle of *ob/ob* mice previously observed [65].

Fiber size augmentation requires an increase of the myofibrillar protein content. FoxO1 down-regulates genes implicated in muscle growth and differentiation [14], and myostatin expression has been shown to be induced by FoxO1 in myotubes and mice [77], [78]. Moreover, both myostatin and FoxO have been shown to inhibit protein synthesis by decreasing the activity of the components of the Akt/mammalian target of rapamycin pathway [70], [79]–[81]. An unexpected finding of our study was the discordance for mRNA and protein expression of myofibrillar MHC and TnT isoforms, which has been suggested to be due to a higher proteolytic rate or lower translational efficiency, rather than changes in the transcription process [82]–[84]. The proteins which are ubiquitinated by MAFbx, MuRF1 and Need4 remain largely unknown, but some evidence indicates that MAFbx ubiquitinates and degradates MyoD [85], Need4 targets Notch [86], and MuRF1 ubiquitinates and degrades Troponin I [87] and MHC [84], through a direct role of FoxO1 on the MuRF1 promoter activity [13]. Our data show that leptin administration prevented the increase of FoxO3a protein activity and MAFbx and MuRF1 gene and protein expression in wild type and *ob/ob* mice. Therefore, the increase of the *Mhc1* and *Mhc2a* and *slow TnT* mRNA levels in *ob/ob* mice in the absence of an increase in their protein levels may represent a compensatory adaptation to increase levels of lost myofibrillar proteins in *ob/ob* mice.

The control of muscle mass is regulated by a dynamic balance between the anabolic and catabolic processes. Data from our study indicate, for the first time, that leptin treatment prevents muscular atrophy by decreasing FoxO3a, MAFbx and MuRF1 protein expression in relation to an increase in the PGC-1α protein in the GAS muscle of wild type and *ob/ob* mice. The reduced skeletal muscle mass associated to leptin deficiency was prevented by leptin, on the one hand, reducing the negative regulator of muscle growth, myostatin, at the same time as by enhancing muscle cell proliferation through an increase in cyclin D1 and PCNA as well as the myofibrillar protein content of TnT, while decreasing p27^Kip1^.

Unfortunately, except in the rare cases of leptin deficiency, the clinical application of leptin in humans has not proved to be worthwhile in common obesity, since most obese patients exhibit hyperleptinemia pointing to the existence of leptin resistance. Therefore, no therapeutic benefit as regards improving body composition in obese humans might be foreseen. Furthermore, the sexual dimorphism relating to the fact that women have smaller muscles in spite of increased circulating leptin concentrations and higher leptin receptors expression should be also contemplated [88]. Nonetheless, practical applications of leptin administration may be envisaged in sports medicine and the area of disuse atrophy, which is a common clinical phenomenon. Prolonged immobilization due to fractures or neuromotor problems may represent scenarios for the application of leptin with therapeutic purposes targeting the restoration of muscle atrophy following limb disuse. Furthermore, from a physiologic perspective researchers involved in sports physiology might be also interested to gain more insight into the regulatory pathways involved in muscle mass accretion. Moreover, our study suggests that leptin treatment may be an attractive therapeutic approach to prevent muscular atrophy associated with catabolic diseases, which might be particularly useful in cachectic patients, such as frequently observed in oncological processes, HIV patients as well as other lipodystrophies.

## Materials and Methods

### Animals and Treatments

Ten-week-old male wild type (C57BL/6J) (n = 30) and genetically obese *ob/ob* mice (C57BL/6J) (n = 30) supplied by Harlan (Barcelona, Spain) were housed in a room with controlled temperature (22±2°C), and a 12∶12 light-dark cycle (lights on at 08:00 am). Wild type and *ob/ob* mice were divided in control, leptin-treated (1 mg/kg/d) and pair-fed groups (n = 10 per group). A pilot study with different leptin doses and routes of administration was carried out in order to select the appropriate dose to ensure that its concentration would fell within the physiological range (nanomolar range) in both the wild type and *ob/ob* groups. The control and pair-fed groups received vehicle (PBS), while leptin-treated groups were intraperitoneally administered with leptin (Bachem, Bubendorf, Switzerland) twice a day at 08:00 and 20:00 for 28 days. Control and leptin-treated groups were provided with water and food *ad libitum* with a standard rodent chow (2014S Teklad, Harlan, Barcelona, Spain), while the daily food intake of the pair-fed groups was matched to the amount eaten by the leptin-treated groups the day before to discriminate the inhibitory effect of leptin on appetite. All experimental procedures conformed to the European Guidelines for the Care and Use of Laboratory Animals (directive 86/609) and were approved by the Ethical Committee for Animal Experimentation of the University of Navarra (080/05). Animals were sacrificed on the 28^th^ day of treatment by CO_2_ inhalation 20 h after the last PBS or leptin administration (in order to avoid picking up effects reflecting an acute response) and after 8 h of fasting. Serum samples were obtained and stored at −80°C. Representative muscles of each muscle fiber type were excised: the SOL muscle is predominantly composed by oxidative, red fibers; the EDL contains mainly glycolytic, white fibers; while the GAS represents a mixed muscle type composed by both red and white fibers. Muscles of one leg were rapidly dissected out, weighed, frozen in liquid nitrogen, and stored at −80°C until mRNA and protein extraction, while the contralateral leg muscles were formalin-fixed for immunohistochemical analyses. Epididymal, perirrenal and subcutaneous adipose tissue depots were also excised.

### Blood Analysis

Serum glucose concentrations were measured using a sensitive-automatic glucose sensor (Ascensia Elite, Bayer, Barcelona, Spain). Serum triglycerides were spectrophotometrically determined using a commercial kit (Infinity, Thermo Electron, Melbourne, Australia). Serum free fatty acid (FFA) concentrations were measured by a colorimetric determination using the NEFA C kit (WAKO Chemicals, Neuss, Germany). Serum glycerol concentrations were evaluated by enzymatic methods [89]. Insulin and leptin were determined using mouse ELISA kits (Crystal Chem Inc., Chicago, IL, USA). Adiponectin and testosterone concentrations were also assessed using ELISA kits (BioVendor Laboratory Medicine, Inc., Modrice, Czech Republic and R&D Systems Europe, Ltd., Abingdon, United Kingdom, respectively). Intra- and inter-assay coefficients of variation for measurements of insulin, leptin, adiponectin and testosterone were 2.6–4.2% for the former, and 5.3–8.1%, for the latter.

### Microarray Experiments and Analysis

Total RNA was extracted from 20–30 mg of GAS muscle samples by homogenization with an ULTRA-TURRAX® T 25 basic (IKA® Werke GmbH, Staufen, Germany) using TRIzol™ reagent (Invitrogen, Barcelona, Spain). Samples were purified using the RNeasy Mini kit (Qiagen, Barcelona, Spain) and treated with DNase I (RNase-free DNase Set, Qiagen) in order to remove any trace of genomic DNA.

Gene expression analyses were conducted using the Agilent Whole Mouse Genome array (G4121B, Agilent Technologies, Santa Clara, CA), containing 41,000 mouse genes and transcripts. Briefly, 1 µg of total RNA from each sample was amino-allyl labeled and amplified using the Amino Allyl MessageAmp II aRNA Amplification Kit (Ambion, Austin, TX, USA). Aliquots (1.2 µg) of amplified aRNA were fluorescently labeled using Cy3/Cy5 (Amersham, Biosciences, Buckinghamshire, UK) and then appropriately combined and hybridized to Agilent oligomicroarrays. Hybridizations were performed following a reference design, where control samples were pools of RNA from all individual samples. Two hybridizations with fluor reversal (Dye swap) were performed for each sample and 5 animals were used per group. After washing, microarray slides were scanned using a Gene Pix 4100A scanner (Axon Instruments, Union City, CA, USA) and image quantization was performed using the software GenePiX Pro 6.0. Gene expression data for all replicate experiments were analyzed using the GeneSpring GX software v 7.3.1 (Agilent Technologies). Clustering was accomplished with the Gene and Condition Tree algorithms. In addition, Gene Ontology groupings (http://babelomics.bioinfo.cipf.es) and the KEGG website (http://www.genome.ad.jp/kegg/pathway) were used in conjunction with GeneSpring (http://www.agilent.com/chem/genespring) to identify pathways and functional groups of genes. All microarray data reported are described in accordance with MIAME guidelines. More information regarding the microarray experiments can be found at the EMBL-European Bioinformatics Institute (http://www.ebi.ac.uk/aerep/login. ArrayExpress accession number: E-MEXP-1831).

### Real-Time PCR

To validate the microarray data, a number of representative differentially expressed genes were selected to be individually studied by Real-Time PCR (n = 5–10 per group). For first strand cDNA synthesis, constant amounts of 1.5 µg of total RNA isolated from GAS muscle were reverse transcribed using random hexamers (Roche Molecular Biochemicals, Mannheim, Germany) as primers and 300 units of M-MLV reverse transcriptase (Invitrogen) as previously described [90]. The transcript levels were quantified by Real-Time PCR (7300 Real Time PCR System, Applied Biosystems, Foster City, CA, USA). Primers and probes were designed using the software Primer Express 2.0 (Applied Biosystems) (Table S4). Probes were designed to hybridize between exons to ensure the detection of the corresponding transcript avoiding genomic DNA amplification. The cDNA was amplified at the following conditions: 95°C for 10 min, followed by 45 cycles of 15 s at 95°C and 1 min at 59°C, using the TaqMan® Universal PCR Master Mix (Applied Biosystems). The primer and probe concentrations for gene amplification were 300 nmoL/L and 200 nmoL/L, respectively. All results were normalized to the levels of *18S* rRNA (Applied Biosystems) and relative quantification was calculated using the ΔΔCt formula [90]. Relative mRNA expression was expressed as fold expression over the calibrator sample (average of gene expression corresponding to the wild type group). All samples were run in triplicate and the average values were calculated.

### Western Blot

Muscle samples (20 mg) were homogenized in RIPA buffer (1 M Tris-HCl pH 7.4, 150 mM NaCl, 1% Triton X-100, 0.1% SDS, EDTA 2H_2_O 5 mM, 1% deoxycolate) supplemented with protease inhibitors (Complete™ Mini-EDTA free, Roche). Soluble proteins were recovered after centrifugation at 16,000 *g* for 15 min at 4°C. Protein concentration was determined according to the method of Bradford [91] using bovine serum albumine (BSA) (Sigma, St. Louis, MO, USA) as standard. Equal amounts of protein (30 µg) were boiled for 10 min and resolved on 10% SDS-PAGE at constant voltage (200 V for 1 h), except for MHC proteins (100 V for 4 h). Then, proteins were transferred to nitrocellulose membranes (BioRad, Hercules, CA, USA) and were blocked with 5% non-fat dry milk in TBS-Tween 20 0.05% for 1 h at room temperature (RT). Blots were then incubated with mouse monoclonal anti-PCNA (Dako Cytomation, Glostrup, Denmark), rabbit polyclonal anti-myostatin, anti-cyclin D1, and anti-p27^Kip1^ (Abcam, Cambridge, UK), mouse polyclonal anti-MHC I, anti-MHC II, goat polyclonal anti-PGC-1α, anti-slow TnT, anti-fast TnT, anti-MAFbx, and rabbit polyclonal anti-MuRF1 (Santa Cruz Biotechnology, Santa Cruz, CA, USA), rabbit polyclonal anti-Akt1/PKB (phospho Thr 308) and anti-Akt1/PKB (Upstate, Cambridge, UK) antibodies in blocking buffer overnight at 4°C. After washing with TBS-Tween 0.5% (5×5 min), membranes were incubated with horseradish peroxidase-conjugated anti-goat IgG (Zymed, San Francisco, CA, USA), anti-rabbit IgG, or anti-mouse IgG (Amersham Biosciences) for 1 h at RT. Mouse monoclonal anti-β-actin (Sigma) was used for normalization of density values. The chemiluminescence ECL reactive (Enhanced Chemiluminescent System, Amersham Biosciences) was used to develop the bands, which were analyzed by densitometric analysis using the Gel Doc-Quantity One 4.5.0. software (Bio-Rad).

### Histological Analyses

Sections (5 µm) of formalin-fixed paraffin-embedded muscles were dewaxed with xylene and hydrated in decreasing concentrations of ethanol. Endogen peroxidase activity was quenched using 3% H_2_O_2_ (Sigma) in absolute methanol for 20 min at RT, and washed 3 times with ethanol. Sections were immersed in 10 mM citrate buffer (pH 6.0) and heated using a microwave oven at 800 W for 10 min to enhance antigen retrieval. After cooling, sections were blocked for 30 min at RT in a humidified chamber with 5% murine or goat serum (Sigma) in TBS. Polyclonal antibodies against MAFbx, MuRF1, PCNA, cyclin D1, p27^Kip1^, fast TnT and slow TnT, used for immunohistochemistry were the same than those used for western blot studies. In addition, the dystrophin protein was also immunolocalized with a monoclonal antibody (Abcam). Sections were subsequently incubated with the appropriate dilutions of primary antibodies in TBS with 5% mouse or goat serum (Sigma) (1∶50–100) in a humidified chamber overnight at 4°C. After washing with TBS (3×5 min), sections were incubated with horseradish peroxidase-conjugated secondary antibodies diluted in TBS with 5% BSA (1∶100) for 1 h at RT. After washing with TBS (3×5 min), localization of the antigen-antibody binding antibodies was performed by adding diaminobenzidine (DAB) (Sigma) or DAB with glucose oxidase (Sigma) as developing system [92]. Negative control slides with omission of the primary antibodies were included in the immunostaining procedure. The reaction was stopped and contrasted with Harris hematoxylin solution (Sigma). Sections were dehydrated with increasing concentrations of ethanol and xylol, mounted in DePeX (Panreac, Barcelona, Spain), and observed with an optic microscope (Axiovert 40 CFL, Zeiss, Göttingen, Germany). Fiber size was determined by measuring the cross-sectional area of GAS, SOL and EDL muscle fibers with the Axiovision 4.6 program (Zeiss). Positive myonuclei for PCNA, cyclin D1 and p27^kip1^ in cross-sections of GAS muscles were quantified from 4 microscope fields (400X) randomly selected. Mean value was expressed as positive myonuclei number per 100 cells.

### Statistical Analysis

Data are expressed as mean±standard error of the mean (SEM). Global effects of genotype and treatment were determined using a two-way analysis of the variance (ANOVA). When interaction between both factors was detected, comparisons between groups were subsequently analyzed by Kruskal-Wallis followed by *U*-Mann Whitney tests. As previously outlined, Gene Ontology groupings were used to identify pathways significantly affected by leptin deficiency and administration. Furthermore, statistical comparisons for microarray data to identify differentially expressed genes across different groups were performed using one-way ANOVA and Student's *t*-tests as appropriate. All statistical analyses were performed by using the SPSS statistical program version 15.0 for Windows (SPSS, Chicago, IL, USA) and statistical significance was defined as *P*<0.05.

## Supporting Information

Table S1 Biochemical Characteristics of Wild Type and ob/ob Mice
(0.03 MB XLS)

Table S2 Genes Differentially Regulated by Leptin Treatment in ob/ob Mice
(0.10 MB XLS)

Table S3 Genes Differentially Regulated by Leptin Treatment as Compared to Pair-Feeding in ob/ob Mice
(0.38 MB XLS)

Table S4 Sequences of the Primers and Taqman Probes Used in the Real-Time PCR
(0.03 MB XLS)

Figure S1 Leptin Treatment Decreases Body Weight and Body Fat in Wild Type and *ob/ob* Mice. (A) Body weight curves of PBS (open), pair-fed (gray) and leptin-treated (closed) wild type and *ob/ob* mice (n = 9–10 animals per group). **P<0.01 and ***P<0.001 for PBS *ob/ob* vs PBS wild type and leptin-treated *ob/ob* mice. +P<0.05, ++P<0.01 and +++P<0.001 for pair-fed *ob/ob* vs leptin-treated *ob/ob*. #P<0.05 and ##P<0.001 for PBS wild type vs leptin-treated wild type. (B) Daily food intake curves of PBS (open), pair-fed (gray) and leptin-treated (closed) wild type and *ob/ob* mice (n = 9–10 animals per group). ***P<0.001 for PBS *ob/ob* vs PBS wild type and leptin-treated *ob/ob*. #P<0.05 and # #P<0.001 for PBS wild type vs leptin-treated wild type. (C) Epididymal (EWAT), perirrenal (PWAT) and subcutaneous (SWAT) depots relative to body weight of PBS (open), pair-fed (gray) and leptin-treated (closed) wild type and *ob/ob* mice (9–10 animals per group). *P<0.05, **P<0.01 and ***P<0.001. Data are presented as mean±SEM. G: genotype, T: treatment. The striped line indicates the beginning of the pair-feeding treatment.
(9.73 MB TIF)

Figure S2 Hierarchical Clustering of the Gene Expression Profile of the Gastrocnemius Muscle of Wild Type, *ob/ob* and Leptin-Treated *ob/ob* Mice. Red represents up-regulated expression, green shows down-regulation, and yellow indicates a similar gene expression pattern as compared to reference. White boxes highlight that leptin treatment was able to reduce the mRNA expression of 732 up-regulated genes in *ob/ob* mice and to increase the expression of 846 down-regulated genes.
(10.13 MB TIF)

Figure S3 Analyses by Real-Time PCR of Key Genes Involved in Muscular Atrophy and Muscle Growth. (A) Real-Time PCR analysis of forkhead box class O3a (Foxo3a) and Foxo1, and peroxisome proliferator-activated receptor coactivator 1α (Pgc-1α) in gastrocnemius muscle of PBS (open), pair-fed (gray) and leptin-treated (closed) wild type and *ob/ob* mice (n = 5 per group). (B) Real-Time PCR analysis of muscle atrophy F box (MAFbx) and muscle RING finger 1 (MuRF1) in gastrocnemius muscle of PBS (open), pair-fed (gray) and leptin-treated (closed) wild type and *ob/ob* mice (n = 5 per group). (C) Real-Time PCR analysis of myosin heavy chain type I (Mhc1), myosin heavy chain type IIa (Mhc2a), myosin heavy chain type IIb (Mhc2b) and slow troponin T (slow TnT), in gastrocnemius muscle of PBS (open), pair-fed (gray) and leptin-treated (closed) wild type and *ob/ob* mice (n = 5 per group). Data are presented as mean±SEM of the ratio between gene expression and 18S rRNA. G: genotype, T: treatment.
(0.39 MB TIF)
