# Supplementary figures and images for: Leptin Administration Favors Muscle Mass Accretion by Decreasing FoxO3a and Increasing PGC-1α in ob/ob Mice
Source: PLoS One. 2009 Sep 4;4(9):e6808. doi: 10.1371/journal.pone.0006808 (PMC2733298; doi:10.1371/journal.pone.0006808)

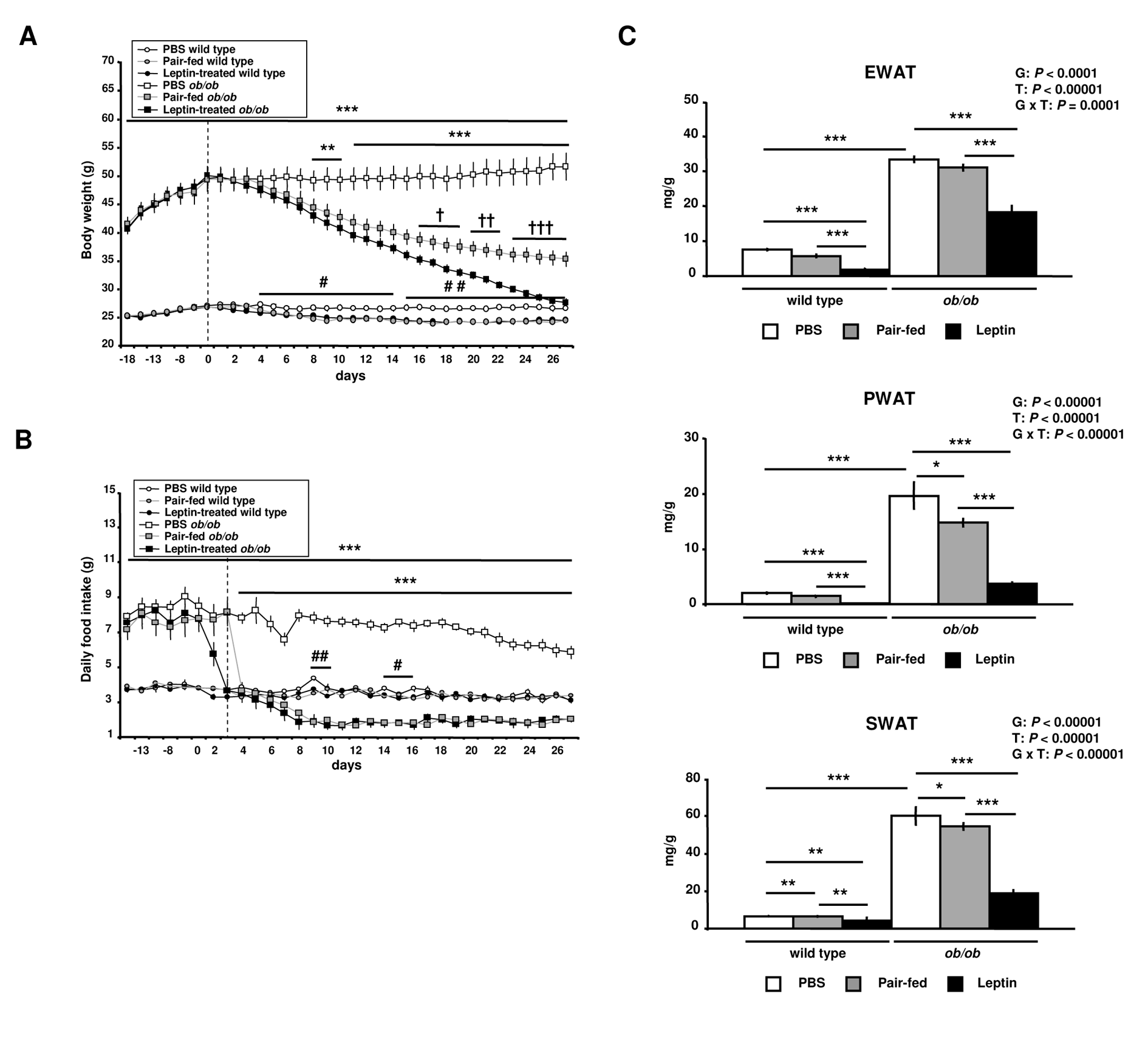

Supplement: Figure S1 — Leptin Treatment Decreases Body Weight and Body Fat in Wild Type and ob/ob Mice. (A) Body weight curves of PBS (open), pair-fed (gray) and leptin-treated (closed) wild type and ob/ob mice (n = 9–10 animals per group). **P<0.01 and ***P<0.001 for PBS ob/ob vs PBS wild type and leptin-treated ob/ob mice. +P<0.05, ++P<0.01 and +++P<0.001 for pair-fed ob/ob vs leptin-treated ob/ob. #P<0.05 and ##P<0.001 for PBS wild type vs leptin-treated wild type. (B) Daily food intake curves of PBS (open), pair-fed (gray) and leptin-treated (closed) wild type and ob/ob mice (n = 9–10 animals per group). ***P<0.001 for PBS ob/ob vs PBS wild type and leptin-treated ob/ob. #P<0.05 and # #P<0.001 for PBS wild type vs leptin-treated wild type. (C) Epididymal (EWAT), perirrenal (PWAT) and subcutaneous (SWAT) depots relative to body weight of PBS (open), pair-fed (gray) and leptin-treated (closed) wild type and ob/ob mice (9–10 animals per group). *P<0.05, **P<0.01 and ***P<0.001. Data are presented as mean±SEM. G: genotype, T: treatment. The striped line indicates the beginning of the pair-feeding treatment. (9.73 MB TIF) [file pone.0006808.s005.tif]

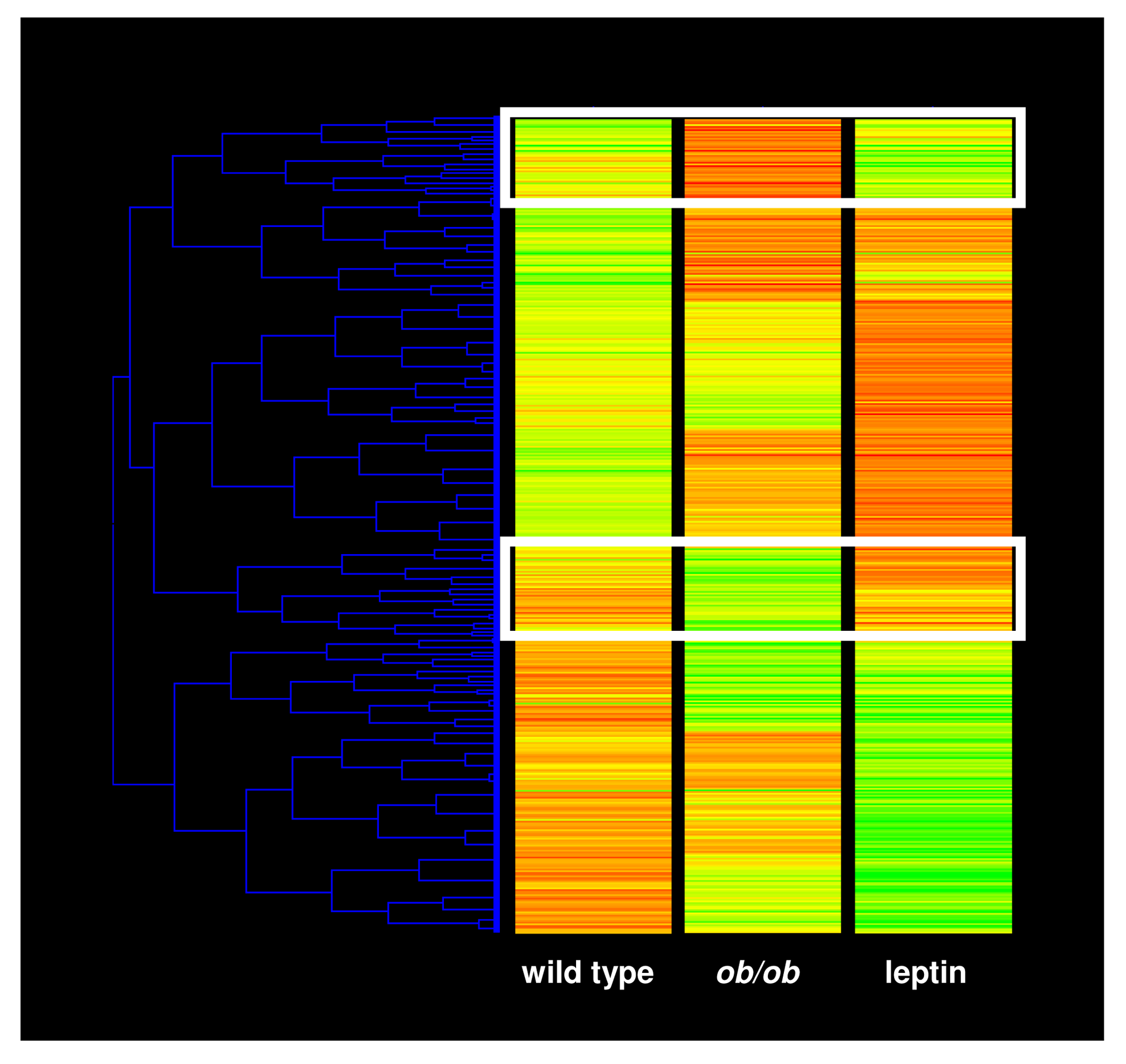

Supplement: Figure S2 — Hierarchical Clustering of the Gene Expression Profile of the Gastrocnemius Muscle of Wild Type, ob/ob and Leptin-Treated ob/ob Mice. Red represents up-regulated expression, green shows down-regulation, and yellow indicates a similar gene expression pattern as compared to reference. White boxes highlight that leptin treatment was able to reduce the mRNA expression of 732 up-regulated genes in ob/ob mice and to increase the expression of 846 down-regulated genes. (10.13 MB TIF) [file pone.0006808.s006.tif]

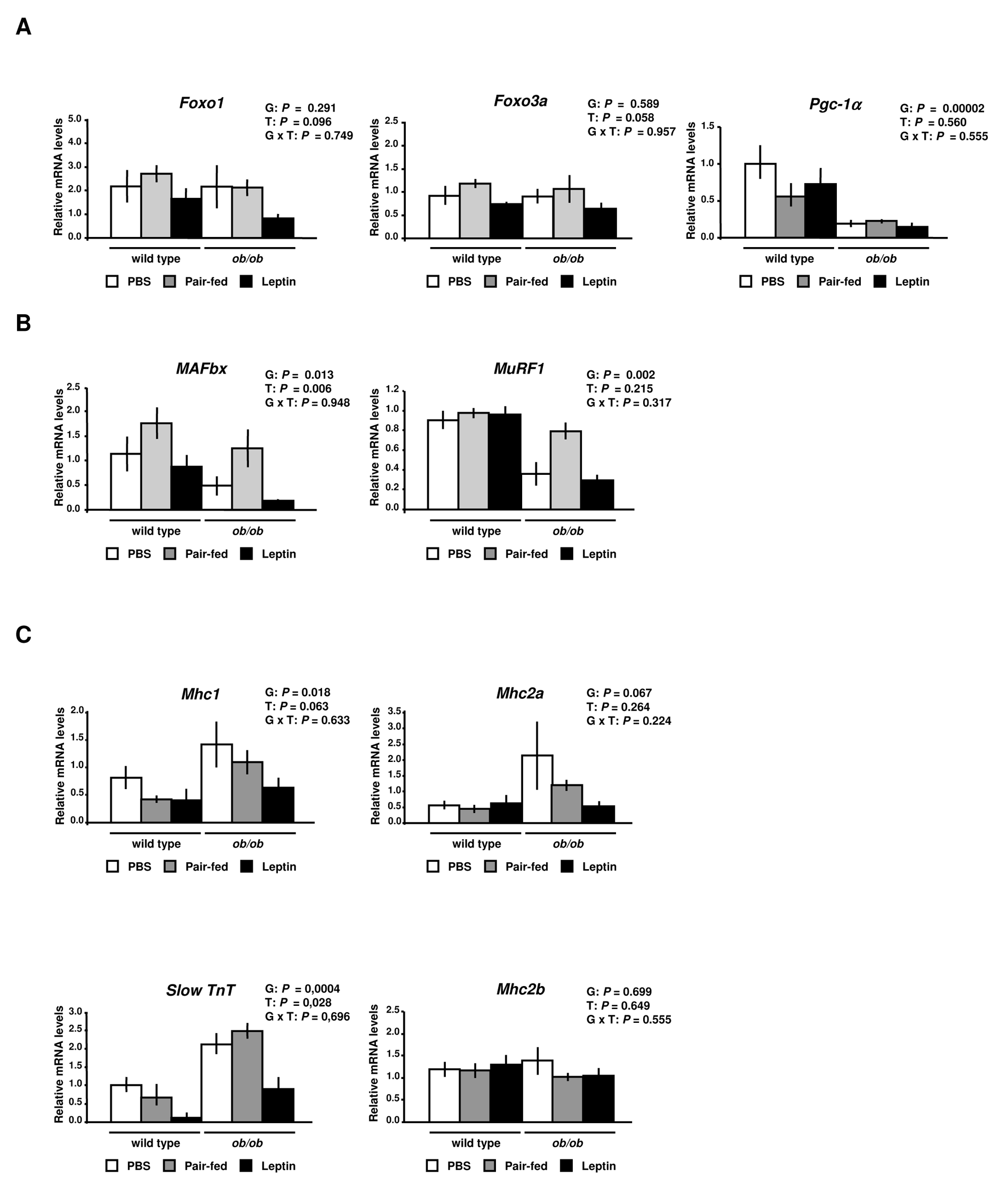

Supplement: Figure S3 — Analyses by Real-Time PCR of Key Genes Involved in Muscular Atrophy and Muscle Growth. (A) Real-Time PCR analysis of forkhead box class O3a (Foxo3a) and Foxo1, and peroxisome proliferator-activated receptor coactivator 1α (Pgc-1α) in gastrocnemius muscle of PBS (open), pair-fed (gray) and leptin-treated (closed) wild type and ob/ob mice (n = 5 per group). (B) Real-Time PCR analysis of muscle atrophy F box (MAFbx) and muscle RING finger 1 (MuRF1) in gastrocnemius muscle of PBS (open), pair-fed (gray) and leptin-treated (closed) wild type and ob/ob mice (n = 5 per group). (C) Real-Time PCR analysis of myosin heavy chain type I (Mhc1), myosin heavy chain type IIa (Mhc2a), myosin heavy chain type IIb (Mhc2b) and slow troponin T (slow TnT), in gastrocnemius muscle of PBS (open), pair-fed (gray) and leptin-treated (closed) wild type and ob/ob mice (n = 5 per group). Data are presented as mean±SEM of the ratio between gene expression and 18S rRNA. G: genotype, T: treatment. (0.39 MB TIF) [file pone.0006808.s007.tif]
